# Supplementary material for: Beyond labs: unveiling dynamics of dental students’ transition from pre-clinical to clinical training in a Saudi dental school
Source: PeerJ. 2024 Sep 10;12:e18019. doi: 10.7717/peerj.18019 (PMC11397122; doi:10.7717/peerj.18019)
Supplement: Supplemental Information 1 [file peerj-12-18019-s001.docx]

| **Descriptives** | | | | |
| --- | --- | --- | --- | --- |
|  | | | Statistic | Std. Error |
| Orientation should be given to all dentistry Students starting the clinics | Mean | | 4.5915 | .06208 |
|  | 95% Confidence Interval for Mean | Lower Bound | 4.4677 |  |
|  |  | Upper Bound | 4.7154 |  |
|  | 5% Trimmed Mean | | 4.6174 |  |
|  | Median | | 5.0000 |  |
|  | Variance | | .274 |  |
|  | Std. Deviation | | .52311 |  |
|  | Minimum | | 3.00 |  |
|  | Maximum | | 5.00 |  |
|  | Range | | 2.00 |  |
|  | Interquartile Range | | 1.00 |  |
|  | Skewness | | -.687 | .285 |
|  | Kurtosis | | -.863 | .563 |
| The first few weeks of clinical work were better than expected | Mean | | 3.8028 | .12139 |
|  | 95% Confidence Interval for Mean | Lower Bound | 3.5607 |  |
|  |  | Upper Bound | 4.0449 |  |
|  | 5% Trimmed Mean | | 3.8678 |  |
|  | Median | | 4.0000 |  |
|  | Variance | | 1.046 |  |
|  | Std. Deviation | | 1.02288 |  |
|  | Minimum | | 1.00 |  |
|  | Maximum | | 5.00 |  |
|  | Range | | 4.00 |  |
|  | Interquartile Range | | 2.00 |  |
|  | Skewness | | -.826 | .285 |
|  | Kurtosis | | .271 | .563 |
| Working with my fellow student was easy | Mean | | 4.1143 | .10484 |
|  | 95% Confidence Interval for Mean | Lower Bound | 3.9051 |  |
|  |  | Upper Bound | 4.3234 |  |
|  | 5% Trimmed Mean | | 4.1825 |  |
|  | Median | | 4.0000 |  |
|  | Variance | | .769 |  |
|  | Std. Deviation | | .87713 |  |
|  | Minimum | | 2.00 |  |
|  | Maximum | | 5.00 |  |
|  | Range | | 3.00 |  |
|  | Interquartile Range | | 1.00 |  |
|  | Skewness | | -.891 | .287 |
|  | Kurtosis | | .291 | .566 |
| I needed time to adjust to the new environment | Mean | | 4.1549 | .09755 |
|  | 95% Confidence Interval for Mean | Lower Bound | 3.9604 |  |
|  |  | Upper Bound | 4.3495 |  |
|  | 5% Trimmed Mean | | 4.2191 |  |
|  | Median | | 4.0000 |  |
|  | Variance | | .676 |  |
|  | Std. Deviation | | .82198 |  |
|  | Minimum | | 2.00 |  |
|  | Maximum | | 5.00 |  |
|  | Range | | 3.00 |  |
|  | Interquartile Range | | 1.00 |  |
|  | Skewness | | -.774 | .285 |
|  | Kurtosis | | .149 | .563 |
| This was the first time I experienced what it is like to work as a dentist | Mean | | 4.1690 | .12200 |
|  | 95% Confidence Interval for Mean | Lower Bound | 3.9257 |  |
|  |  | Upper Bound | 4.4123 |  |
|  | 5% Trimmed Mean | | 4.2746 |  |
|  | Median | | 4.0000 |  |
|  | Variance | | 1.057 |  |
|  | Std. Deviation | | 1.02798 |  |
|  | Minimum | | 1.00 |  |
|  | Maximum | | 5.00 |  |
|  | Range | | 4.00 |  |
|  | Interquartile Range | | 1.00 |  |
|  | Skewness | | -1.243 | .285 |
|  | Kurtosis | | 1.123 | .563 |
| I experienced a great deal of stress | Mean | | 4.1268 | .09813 |
|  | 95% Confidence Interval for Mean | Lower Bound | 3.9310 |  |
|  |  | Upper Bound | 4.3225 |  |
|  | 5% Trimmed Mean | | 4.1721 |  |
|  | Median | | 4.0000 |  |
|  | Variance | | .684 |  |
|  | Std. Deviation | | .82686 |  |
|  | Minimum | | 2.00 |  |
|  | Maximum | | 5.00 |  |
|  | Range | | 3.00 |  |
|  | Interquartile Range | | 1.00 |  |
|  | Skewness | | -.555 | .285 |
|  | Kurtosis | | -.496 | .563 |
| The first few weeks working in the clinics were difficult for me | Mean | | 3.8592 | .11748 |
|  | 95% Confidence Interval for Mean | Lower Bound | 3.6249 |  |
|  |  | Upper Bound | 4.0935 |  |
|  | 5% Trimmed Mean | | 3.9304 |  |
|  | Median | | 4.0000 |  |
|  | Variance | | .980 |  |
|  | Std. Deviation | | .98989 |  |
|  | Minimum | | 1.00 |  |
|  | Maximum | | 5.00 |  |
|  | Range | | 4.00 |  |
|  | Interquartile Range | | 2.00 |  |
|  | Skewness | | -.800 | .285 |
|  | Kurtosis | | .499 | .563 |
| l experienced an abrupt transition from preclinical to clinical training | Mean | | 3.9437 | .10594 |
|  | 95% Confidence Interval for Mean | Lower Bound | 3.7324 |  |
|  |  | Upper Bound | 4.1549 |  |
|  | 5% Trimmed Mean | | 4.0086 |  |
|  | Median | | 4.0000 |  |
|  | Variance | | .797 |  |
|  | Std. Deviation | | .89263 |  |
|  | Minimum | | 1.00 |  |
|  | Maximum | | 5.00 |  |
|  | Range | | 4.00 |  |
|  | Interquartile Range | | 2.00 |  |
|  | Skewness | | -.756 | .285 |
|  | Kurtosis | | .660 | .563 |
| The clinical staff provided sufficient support | Mean | | 3.8406 | .12664 |
|  | 95% Confidence Interval for Mean | Lower Bound | 3.5879 |  |
|  |  | Upper Bound | 4.0933 |  |
|  | 5% Trimmed Mean | | 3.8945 |  |
|  | Median | | 4.0000 |  |
|  | Variance | | 1.107 |  |
|  | Std. Deviation | | 1.05193 |  |
|  | Minimum | | 1.00 |  |
|  | Maximum | | 5.00 |  |
|  | Range | | 4.00 |  |
|  | Interquartile Range | | 2.00 |  |
|  | Skewness | | -.608 | .289 |
|  | Kurtosis | | -.465 | .570 |
| The transition from preclinical to clinical training was smooth | Mean | | 3.8169 | .11750 |
|  | 95% Confidence Interval for Mean | Lower Bound | 3.5826 |  |
|  |  | Upper Bound | 4.0513 |  |
|  | 5% Trimmed Mean | | 3.8678 |  |
|  | Median | | 4.0000 |  |
|  | Variance | | .980 |  |
|  | Std. Deviation | | .99009 |  |
|  | Minimum | | 1.00 |  |
|  | Maximum | | 5.00 |  |
|  | Range | | 4.00 |  |
|  | Interquartile Range | | 2.00 |  |
|  | Skewness | | -.346 | .285 |
|  | Kurtosis | | -.536 | .563 |
| I felt prepared for the clinical training | Mean | | 3.7324 | .13451 |
|  | 95% Confidence Interval for Mean | Lower Bound | 3.4641 |  |
|  |  | Upper Bound | 4.0007 |  |
|  | 5% Trimmed Mean | | 3.8052 |  |
|  | Median | | 4.0000 |  |
|  | Variance | | 1.285 |  |
|  | Std. Deviation | | 1.13336 |  |
|  | Minimum | | 1.00 |  |
|  | Maximum | | 5.00 |  |
|  | Range | | 4.00 |  |
|  | Interquartile Range | | 2.00 |  |
|  | Skewness | | -.661 | .285 |
|  | Kurtosis | | -.322 | .563 |
| I was very uncertain at the beginning of the clinical placements | Mean | | 3.7826 | .13359 |
|  | 95% Confidence Interval for Mean | Lower Bound | 3.5160 |  |
|  |  | Upper Bound | 4.0492 |  |
|  | 5% Trimmed Mean | | 3.8623 |  |
|  | Median | | 4.0000 |  |
|  | Variance | | 1.231 |  |
|  | Std. Deviation | | 1.10971 |  |
|  | Minimum | | 1.00 |  |
|  | Maximum | | 5.00 |  |
|  | Range | | 4.00 |  |
|  | Interquartile Range | | 2.00 |  |
|  | Skewness | | -.684 | .289 |
|  | Kurtosis | | -.113 | .570 |
| I felt prepared for clinical training | Mean | | 3.9143 | .13304 |
|  | 95% Confidence Interval for Mean | Lower Bound | 3.6489 |  |
|  |  | Upper Bound | 4.1797 |  |
|  | 5% Trimmed Mean | | 4.0079 |  |
|  | Median | | 4.0000 |  |
|  | Variance | | 1.239 |  |
|  | Std. Deviation | | 1.11307 |  |
|  | Minimum | | 1.00 |  |
|  | Maximum | | 5.00 |  |
|  | Range | | 4.00 |  |
|  | Interquartile Range | | 2.00 |  |
|  | Skewness | | -.930 | .287 |
|  | Kurtosis | | .251 | .566 |
| The workload during the clinics is heavy | Mean | | 3.9429 | .11491 |
|  | 95% Confidence Interval for Mean | Lower Bound | 3.7136 |  |
|  |  | Upper Bound | 4.1721 |  |
|  | 5% Trimmed Mean | | 4.0079 |  |
|  | Median | | 4.0000 |  |
|  | Variance | | .924 |  |
|  | Std. Deviation | | .96137 |  |
|  | Minimum | | 1.00 |  |
|  | Maximum | | 5.00 |  |
|  | Range | | 4.00 |  |
|  | Interquartile Range | | 2.00 |  |
|  | Skewness | | -.790 | .287 |
|  | Kurtosis | | .270 | .566 |
| There is a significant difference between my workload before and after the transition into the clinical training | Mean | | 4.0282 | .10024 |
|  | 95% Confidence Interval for Mean | Lower Bound | 3.8282 |  |
|  |  | Upper Bound | 4.2281 |  |
|  | 5% Trimmed Mean | | 4.0782 |  |
|  | Median | | 4.0000 |  |
|  | Variance | | .713 |  |
|  | Std. Deviation | | .84468 |  |
|  | Minimum | | 1.00 |  |
|  | Maximum | | 5.00 |  |
|  | Range | | 4.00 |  |
|  | Interquartile Range | | 1.00 |  |
|  | Skewness | | -.786 | .285 |
|  | Kurtosis | | 1.068 | .563 |
| Clinical workload is tiring | Mean | | 3.7857 | .11310 |
|  | 95% Confidence Interval for Mean | Lower Bound | 3.5601 |  |
|  |  | Upper Bound | 4.0113 |  |
|  | 5% Trimmed Mean | | 3.8492 |  |
|  | Median | | 4.0000 |  |
|  | Variance | | .895 |  |
|  | Std. Deviation | | .94628 |  |
|  | Minimum | | 1.00 |  |
|  | Maximum | | 5.00 |  |
|  | Range | | 4.00 |  |
|  | Interquartile Range | | 1.25 |  |
|  | Skewness | | -.609 | .287 |
|  | Kurtosis | | .536 | .566 |
| Clinical work hours are very long | Mean | | 3.5362 | .14390 |
|  | 95% Confidence Interval for Mean | Lower Bound | 3.2491 |  |
|  |  | Upper Bound | 3.8234 |  |
|  | 5% Trimmed Mean | | 3.5958 |  |
|  | Median | | 4.0000 |  |
|  | Variance | | 1.429 |  |
|  | Std. Deviation | | 1.19533 |  |
|  | Minimum | | 1.00 |  |
|  | Maximum | | 5.00 |  |
|  | Range | | 4.00 |  |
|  | Interquartile Range | | 2.00 |  |
|  | Skewness | | -.407 | .289 |
|  | Kurtosis | | -.728 | .570 |
| It was difficult getting used to the work routine | Mean | | 3.6761 | .13272 |
|  | 95% Confidence Interval for Mean | Lower Bound | 3.4113 |  |
|  |  | Upper Bound | 3.9408 |  |
|  | 5% Trimmed Mean | | 3.7426 |  |
|  | Median | | 4.0000 |  |
|  | Variance | | 1.251 |  |
|  | Std. Deviation | | 1.11835 |  |
|  | Minimum | | 1.00 |  |
|  | Maximum | | 5.00 |  |
|  | Range | | 4.00 |  |
|  | Interquartile Range | | 2.00 |  |
|  | Skewness | | -.583 | .285 |
|  | Kurtosis | | -.355 | .563 |
| I get sufficient time to study during my clinical rotations | Mean | | 3.4366 | .12645 |
|  | 95% Confidence Interval for Mean | Lower Bound | 3.1844 |  |
|  |  | Upper Bound | 3.6888 |  |
|  | 5% Trimmed Mean | | 3.4765 |  |
|  | Median | | 3.0000 |  |
|  | Variance | | 1.135 |  |
|  | Std. Deviation | | 1.06546 |  |
|  | Minimum | | 1.00 |  |
|  | Maximum | | 5.00 |  |
|  | Range | | 4.00 |  |
|  | Interquartile Range | | 1.00 |  |
|  | Skewness | | -.304 | .285 |
|  | Kurtosis | | -.437 | .563 |
| Contact with real patients is easy for me | Mean | | 4.0000 | .11348 |
|  | 95% Confidence Interval for Mean | Lower Bound | 3.7737 |  |
|  |  | Upper Bound | 4.2263 |  |
|  | 5% Trimmed Mean | | 4.0712 |  |
|  | Median | | 4.0000 |  |
|  | Variance | | .914 |  |
|  | Std. Deviation | | .95618 |  |
|  | Minimum | | 1.00 |  |
|  | Maximum | | 5.00 |  |
|  | Range | | 4.00 |  |
|  | Interquartile Range | | 1.00 |  |
|  | Skewness | | -1.009 | .285 |
|  | Kurtosis | | .740 | .563 |
| Contact with real patients stimulates me to study | Mean | | 4.0986 | .09859 |
|  | 95% Confidence Interval for Mean | Lower Bound | 3.9020 |  |
|  |  | Upper Bound | 4.2952 |  |
|  | 5% Trimmed Mean | | 4.1565 |  |
|  | Median | | 4.0000 |  |
|  | Variance | | .690 |  |
|  | Std. Deviation | | .83075 |  |
|  | Minimum | | 2.00 |  |
|  | Maximum | | 5.00 |  |
|  | Range | | 3.00 |  |
|  | Interquartile Range | | 1.00 |  |
|  | Skewness | | -.650 | .285 |
|  | Kurtosis | | -.121 | .563 |
| My first contact with real patients was during the first semester of third year | Mean | | 4.0714 | .10636 |
|  | 95% Confidence Interval for Mean | Lower Bound | 3.8592 |  |
|  |  | Upper Bound | 4.2836 |  |
|  | 5% Trimmed Mean | | 4.1349 |  |
|  | Median | | 4.0000 |  |
|  | Variance | | .792 |  |
|  | Std. Deviation | | .88990 |  |
|  | Minimum | | 2.00 |  |
|  | Maximum | | 5.00 |  |
|  | Range | | 3.00 |  |
|  | Interquartile Range | | 1.00 |  |
|  | Skewness | | -.778 | .287 |
|  | Kurtosis | | -.014 | .566 |
| I would have liked real patient contact earlier in the dental program | Mean | | 3.8732 | .12355 |
|  | 95% Confidence Interval for Mean | Lower Bound | 3.6268 |  |
|  |  | Upper Bound | 4.1196 |  |
|  | 5% Trimmed Mean | | 3.9304 |  |
|  | Median | | 4.0000 |  |
|  | Variance | | 1.084 |  |
|  | Std. Deviation | | 1.04101 |  |
|  | Minimum | | 1.00 |  |
|  | Maximum | | 5.00 |  |
|  | Range | | 4.00 |  |
|  | Interquartile Range | | 2.00 |  |
|  | Skewness | | -.521 | .285 |
|  | Kurtosis | | -.570 | .563 |
| I feel patients feel uncomfortable when they are examined by a student | Mean | | 3.6056 | .14253 |
|  | 95% Confidence Interval for Mean | Lower Bound | 3.3214 |  |
|  |  | Upper Bound | 3.8899 |  |
|  | 5% Trimmed Mean | | 3.6729 |  |
|  | Median | | 4.0000 |  |
|  | Variance | | 1.442 |  |
|  | Std. Deviation | | 1.20094 |  |
|  | Minimum | | 1.00 |  |
|  | Maximum | | 5.00 |  |
|  | Range | | 4.00 |  |
|  | Interquartile Range | | 2.00 |  |
|  | Skewness | | -.664 | .285 |
|  | Kurtosis | | -.434 | .563 |
| I feel uncomfortable when I examine a patient. | Mean | | 3.0704 | .15709 |
|  | 95% Confidence Interval for Mean | Lower Bound | 2.7571 |  |
|  |  | Upper Bound | 3.3837 |  |
|  | 5% Trimmed Mean | | 3.0782 |  |
|  | Median | | 3.0000 |  |
|  | Variance | | 1.752 |  |
|  | Std. Deviation | | 1.32367 |  |
|  | Minimum | | 1.00 |  |
|  | Maximum | | 5.00 |  |
|  | Range | | 4.00 |  |
|  | Interquartile Range | | 2.00 |  |
|  | Skewness | | -.095 | .285 |
|  | Kurtosis | | -1.107 | .563 |
| I can perform dental examination ( extra and intraoral ) | Mean | | 4.0857 | .09489 |
|  | 95% Confidence Interval for Mean | Lower Bound | 3.8964 |  |
|  |  | Upper Bound | 4.2750 |  |
|  | 5% Trimmed Mean | | 4.1429 |  |
|  | Median | | 4.0000 |  |
|  | Variance | | .630 |  |
|  | Std. Deviation | | .79387 |  |
|  | Minimum | | 2.00 |  |
|  | Maximum | | 5.00 |  |
|  | Range | | 3.00 |  |
|  | Interquartile Range | | 1.00 |  |
|  | Skewness | | -.693 | .287 |
|  | Kurtosis | | .290 | .566 |
| Clinical faculty supervises while I examine the patient | Mean | | 3.7887 | .10787 |
|  | 95% Confidence Interval for Mean | Lower Bound | 3.5736 |  |
|  |  | Upper Bound | 4.0039 |  |
|  | 5% Trimmed Mean | | 3.8365 |  |
|  | Median | | 4.0000 |  |
|  | Variance | | .826 |  |
|  | Std. Deviation | | .90893 |  |
|  | Minimum | | 1.00 |  |
|  | Maximum | | 5.00 |  |
|  | Range | | 4.00 |  |
|  | Interquartile Range | | 1.00 |  |
|  | Skewness | | -.620 | .285 |
|  | Kurtosis | | .301 | .563 |
| I can take history of the patient | Mean | | 4.1714 | .09094 |
|  | 95% Confidence Interval for Mean | Lower Bound | 3.9900 |  |
|  |  | Upper Bound | 4.3528 |  |
|  | 5% Trimmed Mean | | 4.1905 |  |
|  | Median | | 4.0000 |  |
|  | Variance | | .579 |  |
|  | Std. Deviation | | .76084 |  |
|  | Minimum | | 3.00 |  |
|  | Maximum | | 5.00 |  |
|  | Range | | 2.00 |  |
|  | Interquartile Range | | 1.00 |  |
|  | Skewness | | -.301 | .287 |
|  | Kurtosis | | -1.201 | .566 |
| I can do an oral health assessment | Mean | | 3.9859 | .11951 |
|  | 95% Confidence Interval for Mean | Lower Bound | 3.7476 |  |
|  |  | Upper Bound | 4.2243 |  |
|  | 5% Trimmed Mean | | 4.0712 |  |
|  | Median | | 4.0000 |  |
|  | Variance | | 1.014 |  |
|  | Std. Deviation | | 1.00702 |  |
|  | Minimum | | 1.00 |  |
|  | Maximum | | 5.00 |  |
|  | Range | | 4.00 |  |
|  | Interquartile Range | | 2.00 |  |
|  | Skewness | | -1.008 | .285 |
|  | Kurtosis | | .787 | .563 |
| I can communicate comfortably with the patient | Mean | | 4.2676 | .09400 |
|  | 95% Confidence Interval for Mean | Lower Bound | 4.0801 |  |
|  |  | Upper Bound | 4.4551 |  |
|  | 5% Trimmed Mean | | 4.3130 |  |
|  | Median | | 4.0000 |  |
|  | Variance | | .627 |  |
|  | Std. Deviation | | .79206 |  |
|  | Minimum | | 2.00 |  |
|  | Maximum | | 5.00 |  |
|  | Range | | 3.00 |  |
|  | Interquartile Range | | 1.00 |  |
|  | Skewness | | -.699 | .285 |
|  | Kurtosis | | -.482 | .563 |
| I can apply knowledge in practice | Mean | | 4.2754 | .09206 |
|  | 95% Confidence Interval for Mean | Lower Bound | 4.0917 |  |
|  |  | Upper Bound | 4.4591 |  |
|  | 5% Trimmed Mean | | 4.3382 |  |
|  | Median | | 4.0000 |  |
|  | Variance | | .585 |  |
|  | Std. Deviation | | .76474 |  |
|  | Minimum | | 2.00 |  |
|  | Maximum | | 5.00 |  |
|  | Range | | 3.00 |  |
|  | Interquartile Range | | 1.00 |  |
|  | Skewness | | -.924 | .289 |
|  | Kurtosis | | .647 | .570 |
| The knowledge acquired during preclinical training is applied in the clinical phase | Mean | | 4.0845 | .09355 |
|  | 95% Confidence Interval for Mean | Lower Bound | 3.8979 |  |
|  |  | Upper Bound | 4.2711 |  |
|  | 5% Trimmed Mean | | 4.1252 |  |
|  | Median | | 4.0000 |  |
|  | Variance | | .621 |  |
|  | Std. Deviation | | .78824 |  |
|  | Minimum | | 2.00 |  |
|  | Maximum | | 5.00 |  |
|  | Range | | 3.00 |  |
|  | Interquartile Range | | 1.00 |  |
|  | Skewness | | -.512 | .285 |
|  | Kurtosis | | -.234 | .563 |
| I feel there are gaps in my knowledge | Mean | | 3.7606 | .11777 |
|  | 95% Confidence Interval for Mean | Lower Bound | 3.5257 |  |
|  |  | Upper Bound | 3.9954 |  |
|  | 5% Trimmed Mean | | 3.8052 |  |
|  | Median | | 4.0000 |  |
|  | Variance | | .985 |  |
|  | Std. Deviation | | .99232 |  |
|  | Minimum | | 1.00 |  |
|  | Maximum | | 5.00 |  |
|  | Range | | 4.00 |  |
|  | Interquartile Range | | 2.00 |  |
|  | Skewness | | -.399 | .285 |
|  | Kurtosis | | -.442 | .563 |
| I have sufficient basic science knowledge | Mean | | 3.9714 | .09963 |
|  | 95% Confidence Interval for Mean | Lower Bound | 3.7727 |  |
|  |  | Upper Bound | 4.1702 |  |
|  | 5% Trimmed Mean | | 4.0159 |  |
|  | Median | | 4.0000 |  |
|  | Variance | | .695 |  |
|  | Std. Deviation | | .83356 |  |
|  | Minimum | | 1.00 |  |
|  | Maximum | | 5.00 |  |
|  | Range | | 4.00 |  |
|  | Interquartile Range | | 2.00 |  |
|  | Skewness | | -.718 | .287 |
|  | Kurtosis | | 1.076 | .566 |
| I am prepared to perform clinical skills | Mean | | 4.1127 | .09094 |
|  | 95% Confidence Interval for Mean | Lower Bound | 3.9313 |  |
|  |  | Upper Bound | 4.2940 |  |
|  | 5% Trimmed Mean | | 4.1408 |  |
|  | Median | | 4.0000 |  |
|  | Variance | | .587 |  |
|  | Std. Deviation | | .76624 |  |
|  | Minimum | | 2.00 |  |
|  | Maximum | | 5.00 |  |
|  | Range | | 3.00 |  |
|  | Interquartile Range | | 1.00 |  |
|  | Skewness | | -.392 | .285 |
|  | Kurtosis | | -.593 | .563 |
| The knowledge required in clinical practice is different from my theoretical knowledge | Mean | | 3.8028 | .12304 |
|  | 95% Confidence Interval for Mean | Lower Bound | 3.5574 |  |
|  |  | Upper Bound | 4.0482 |  |
|  | 5% Trimmed Mean | | 3.8521 |  |
|  | Median | | 4.0000 |  |
|  | Variance | | 1.075 |  |
|  | Std. Deviation | | 1.03675 |  |
|  | Minimum | | 1.00 |  |
|  | Maximum | | 5.00 |  |
|  | Range | | 4.00 |  |
|  | Interquartile Range | | 2.00 |  |
|  | Skewness | | -.540 | .285 |
|  | Kurtosis | | -.492 | .563 |
| I have sufficient clinical science knowledge. | Mean | | 3.9437 | .08718 |
|  | 95% Confidence Interval for Mean | Lower Bound | 3.7698 |  |
|  |  | Upper Bound | 4.1175 |  |
|  | 5% Trimmed Mean | | 3.9531 |  |
|  | Median | | 4.0000 |  |
|  | Variance | | .540 |  |
|  | Std. Deviation | | .73460 |  |
|  | Minimum | | 2.00 |  |
|  | Maximum | | 5.00 |  |
|  | Range | | 3.00 |  |
|  | Interquartile Range | | 1.00 |  |
|  | Skewness | | -.134 | .285 |
|  | Kurtosis | | -.542 | .563 |
| I have sufficient behavioral science knowledge. | Mean | | 3.8592 | .09472 |
|  | 95% Confidence Interval for Mean | Lower Bound | 3.6702 |  |
|  |  | Upper Bound | 4.0481 |  |
|  | 5% Trimmed Mean | | 3.8748 |  |
|  | Median | | 4.0000 |  |
|  | Variance | | .637 |  |
|  | Std. Deviation | | .79814 |  |
|  | Minimum | | 2.00 |  |
|  | Maximum | | 5.00 |  |
|  | Range | | 3.00 |  |
|  | Interquartile Range | | 1.00 |  |
|  | Skewness | | -.086 | .285 |
|  | Kurtosis | | -.717 | .563 |
| The level of my knowledge is sufficient | Mean | | 3.7183 | .10067 |
|  | 95% Confidence Interval for Mean | Lower Bound | 3.5175 |  |
|  |  | Upper Bound | 3.9191 |  |
|  | 5% Trimmed Mean | | 3.7426 |  |
|  | Median | | 4.0000 |  |
|  | Variance | | .720 |  |
|  | Std. Deviation | | .84824 |  |
|  | Minimum | | 2.00 |  |
|  | Maximum | | 5.00 |  |
|  | Range | | 3.00 |  |
|  | Interquartile Range | | 1.00 |  |
|  | Skewness | | .004 | .285 |
|  | Kurtosis | | -.736 | .563 |
| I felt prepared for clinical skill performance | Mean | | 3.9286 | .11019 |
|  | 95% Confidence Interval for Mean | Lower Bound | 3.7088 |  |
|  |  | Upper Bound | 4.1484 |  |
|  | 5% Trimmed Mean | | 3.9921 |  |
|  | Median | | 4.0000 |  |
|  | Variance | | .850 |  |
|  | Std. Deviation | | .92190 |  |
|  | Minimum | | 1.00 |  |
|  | Maximum | | 5.00 |  |
|  | Range | | 4.00 |  |
|  | Interquartile Range | | 2.00 |  |
|  | Skewness | | -.655 | .287 |
|  | Kurtosis | | .266 | .566 |
| There is a lot of learning from chairside teaching | Mean | | 4.2113 | .10407 |
|  | 95% Confidence Interval for Mean | Lower Bound | 4.0037 |  |
|  |  | Upper Bound | 4.4188 |  |
|  | 5% Trimmed Mean | | 4.2973 |  |
|  | Median | | 4.0000 |  |
|  | Variance | | .769 |  |
|  | Std. Deviation | | .87693 |  |
|  | Minimum | | 1.00 |  |
|  | Maximum | | 5.00 |  |
|  | Range | | 4.00 |  |
|  | Interquartile Range | | 1.00 |  |
|  | Skewness | | -1.216 | .285 |
|  | Kurtosis | | 1.751 | .563 |
| The knowledge acquired in clinical is easier to remember | Mean | | 4.2113 | .09603 |
|  | 95% Confidence Interval for Mean | Lower Bound | 4.0197 |  |
|  |  | Upper Bound | 4.4028 |  |
|  | 5% Trimmed Mean | | 4.2660 |  |
|  | Median | | 4.0000 |  |
|  | Variance | | .655 |  |
|  | Std. Deviation | | .80915 |  |
|  | Minimum | | 2.00 |  |
|  | Maximum | | 5.00 |  |
|  | Range | | 3.00 |  |
|  | Interquartile Range | | 1.00 |  |
|  | Skewness | | -.741 | .285 |
|  | Kurtosis | | -.113 | .563 |
| Clinical faculty are good teachers | Mean | | 4.1408 | .09682 |
|  | 95% Confidence Interval for Mean | Lower Bound | 3.9477 |  |
|  |  | Upper Bound | 4.3340 |  |
|  | 5% Trimmed Mean | | 4.1878 |  |
|  | Median | | 4.0000 |  |
|  | Variance | | .666 |  |
|  | Std. Deviation | | .81584 |  |
|  | Minimum | | 2.00 |  |
|  | Maximum | | 5.00 |  |
|  | Range | | 3.00 |  |
|  | Interquartile Range | | 1.00 |  |
|  | Skewness | | -.592 | .285 |
|  | Kurtosis | | -.366 | .563 |
| I am able to judge my own progress | Mean | | 4.0423 | .10117 |
|  | 95% Confidence Interval for Mean | Lower Bound | 3.8405 |  |
|  |  | Upper Bound | 4.2440 |  |
|  | 5% Trimmed Mean | | 4.1025 |  |
|  | Median | | 4.0000 |  |
|  | Variance | | .727 |  |
|  | Std. Deviation | | .85250 |  |
|  | Minimum | | 2.00 |  |
|  | Maximum | | 5.00 |  |
|  | Range | | 3.00 |  |
|  | Interquartile Range | | 1.00 |  |
|  | Skewness | | -.651 | .285 |
|  | Kurtosis | | -.095 | .563 |
| I study primarily to pass tests and examinations | Mean | | 3.9155 | .12961 |
|  | 95% Confidence Interval for Mean | Lower Bound | 3.6570 |  |
|  |  | Upper Bound | 4.1740 |  |
|  | 5% Trimmed Mean | | 3.9930 |  |
|  | Median | | 4.0000 |  |
|  | Variance | | 1.193 |  |
|  | Std. Deviation | | 1.09213 |  |
|  | Minimum | | 1.00 |  |
|  | Maximum | | 5.00 |  |
|  | Range | | 4.00 |  |
|  | Interquartile Range | | 2.00 |  |
|  | Skewness | | -.844 | .285 |
|  | Kurtosis | | -.035 | .563 |
| What I study depends on the problems I face during my clinical exposure | Mean | | 3.8310 | .09618 |
|  | 95% Confidence Interval for Mean | Lower Bound | 3.6392 |  |
|  |  | Upper Bound | 4.0228 |  |
|  | 5% Trimmed Mean | | 3.8748 |  |
|  | Median | | 4.0000 |  |
|  | Variance | | .657 |  |
|  | Std. Deviation | | .81040 |  |
|  | Minimum | | 1.00 |  |
|  | Maximum | | 5.00 |  |
|  | Range | | 4.00 |  |
|  | Interquartile Range | | 1.00 |  |
|  | Skewness | | -.672 | .285 |
|  | Kurtosis | | 1.183 | .563 |
| Problem based learning ( PBL ) provided good preparation for clinical practice | Mean | | 3.5352 | .12974 |
|  | 95% Confidence Interval for Mean | Lower Bound | 3.2764 |  |
|  |  | Upper Bound | 3.7940 |  |
|  | 5% Trimmed Mean | | 3.5947 |  |
|  | Median | | 4.0000 |  |
|  | Variance | | 1.195 |  |
|  | Std. Deviation | | 1.09324 |  |
|  | Minimum | | 1.00 |  |
|  | Maximum | | 5.00 |  |
|  | Range | | 4.00 |  |
|  | Interquartile Range | | 1.00 |  |
|  | Skewness | | -.666 | .285 |
|  | Kurtosis | | .105 | .563 |
| I study more after starting my clinical training | Mean | | 3.8732 | .11685 |
|  | 95% Confidence Interval for Mean | Lower Bound | 3.6402 |  |
|  |  | Upper Bound | 4.1063 |  |
|  | 5% Trimmed Mean | | 3.9460 |  |
|  | Median | | 4.0000 |  |
|  | Variance | | .969 |  |
|  | Std. Deviation | | .98459 |  |
|  | Minimum | | 1.00 |  |
|  | Maximum | | 5.00 |  |
|  | Range | | 4.00 |  |
|  | Interquartile Range | | 2.00 |  |
|  | Skewness | | -.940 | .285 |
|  | Kurtosis | | .767 | .563 |
| My learning is influenced by the questions from clinical faculty | Mean | | 3.7746 | .10652 |
|  | 95% Confidence Interval for Mean | Lower Bound | 3.5622 |  |
|  |  | Upper Bound | 3.9871 |  |
|  | 5% Trimmed Mean | | 3.8365 |  |
|  | Median | | 4.0000 |  |
|  | Variance | | .806 |  |
|  | Std. Deviation | | .89757 |  |
|  | Minimum | | 1.00 |  |
|  | Maximum | | 5.00 |  |
|  | Range | | 4.00 |  |
|  | Interquartile Range | | 1.00 |  |
|  | Skewness | | -.875 | .285 |
|  | Kurtosis | | 1.275 | .563 |
| I feel the need to study because I have forgotten my theoretical knowledge | Mean | | 3.8451 | .12631 |
|  | 95% Confidence Interval for Mean | Lower Bound | 3.5931 |  |
|  |  | Upper Bound | 4.0970 |  |
|  | 5% Trimmed Mean | | 3.9304 |  |
|  | Median | | 4.0000 |  |
|  | Variance | | 1.133 |  |
|  | Std. Deviation | | 1.06433 |  |
|  | Minimum | | 1.00 |  |
|  | Maximum | | 5.00 |  |
|  | Range | | 4.00 |  |
|  | Interquartile Range | | 2.00 |  |
|  | Skewness | | -.779 | .285 |
|  | Kurtosis | | .287 | .563 |
| Pre-clinical courses were good preparation for learning skills which are applied in the clinics | Mean | | 3.9714 | .10169 |
|  | 95% Confidence Interval for Mean | Lower Bound | 3.7686 |  |
|  |  | Upper Bound | 4.1743 |  |
|  | 5% Trimmed Mean | | 4.0159 |  |
|  | Median | | 4.0000 |  |
|  | Variance | | .724 |  |
|  | Std. Deviation | | .85077 |  |
|  | Minimum | | 2.00 |  |
|  | Maximum | | 5.00 |  |
|  | Range | | 3.00 |  |
|  | Interquartile Range | | 2.00 |  |
|  | Skewness | | -.381 | .287 |
|  | Kurtosis | | -.601 | .566 |

| **Tests of Normality** | | | | | | |
| --- | --- | --- | --- | --- | --- | --- |
|  | Kolmogorov-Smirnov^a^ | | | Shapiro-Wilk | | |
|  | Statistic | df | Sig. | Statistic | df | Sig. |
| Orientation should be given to all dentistry Students starting the clinics | .388 | 71 | .000 | .659 | 71 | .000 |
| The first few weeks of clinical work were better than expected | .281 | 71 | .000 | .855 | 71 | .000 |
| Working with my fellow student was easy | .262 | 70 | .000 | .807 | 70 | .000 |
| I needed time to adjust to the new environment | .242 | 71 | .000 | .815 | 71 | .000 |
| This was the first time I experienced what it is like to work as a dentist | .284 | 71 | .000 | .777 | 71 | .000 |
| I experienced a great deal of stress | .235 | 71 | .000 | .826 | 71 | .000 |
| The first few weeks working in the clinics were difficult for me | .247 | 71 | .000 | .859 | 71 | .000 |
| l experienced an abrupt transition from preclinical to clinical training | .258 | 71 | .000 | .850 | 71 | .000 |
| The clinical staff provided sufficient support | .227 | 69 | .000 | .863 | 69 | .000 |
| The transition from preclinical to clinical training was smooth | .204 | 71 | .000 | .862 | 71 | .000 |
| I felt prepared for the clinical training | .227 | 71 | .000 | .872 | 71 | .000 |
| I was very uncertain at the beginning of the clinical placements | .201 | 69 | .000 | .866 | 69 | .000 |
| I felt prepared for clinical training | .231 | 70 | .000 | .837 | 70 | .000 |
| The workload during the clinics is heavy | .252 | 70 | .000 | .851 | 70 | .000 |
| There is a significant difference between my workload before and after the transition into the clinical training | .247 | 71 | .000 | .832 | 71 | .000 |
| Clinical workload is tiring | .218 | 70 | .000 | .862 | 70 | .000 |
| Clinical work hours are very long | .187 | 69 | .000 | .892 | 69 | .000 |
| It was difficult getting used to the work routine | .220 | 71 | .000 | .882 | 71 | .000 |
| I get sufficient time to study during my clinical rotations | .194 | 71 | .000 | .906 | 71 | .000 |
| Contact with real patients is easy for me | .289 | 71 | .000 | .819 | 71 | .000 |
| Contact with real patients stimulates me to study | .241 | 71 | .000 | .829 | 71 | .000 |
| My first contact with real patients was during the first semester of third year | .254 | 70 | .000 | .823 | 70 | .000 |
| I would have liked real patient contact earlier in the dental program | .213 | 71 | .000 | .859 | 71 | .000 |
| I feel patients feel uncomfortable when they are examined by a student | .248 | 71 | .000 | .872 | 71 | .000 |
| I feel uncomfortable when I examine a patient. | .167 | 71 | .000 | .904 | 71 | .000 |
| I can perform dental examination ( extra and intraoral ) | .271 | 70 | .000 | .821 | 70 | .000 |
| Clinical faculty supervises while I examine the patient | .268 | 71 | .000 | .867 | 71 | .000 |
| I can take history of the patient | .248 | 70 | .000 | .796 | 70 | .000 |
| I can do an oral health assessment | .252 | 71 | .000 | .832 | 71 | .000 |
| I can communicate comfortably with the patient | .287 | 71 | .000 | .791 | 71 | .000 |
| I can apply knowledge in practice | .263 | 69 | .000 | .785 | 69 | .000 |
| The knowledge acquired during preclinical training is applied in the clinical phase | .246 | 71 | .000 | .832 | 71 | .000 |
| I feel there are gaps in my knowledge | .201 | 71 | .000 | .881 | 71 | .000 |
| I have sufficient basic science knowledge | .257 | 70 | .000 | .837 | 70 | .000 |
| I am prepared to perform clinical skills | .230 | 71 | .000 | .826 | 71 | .000 |
| The knowledge required in clinical practice is different from my theoretical knowledge | .223 | 71 | .000 | .872 | 71 | .000 |
| I have sufficient clinical science knowledge. | .263 | 71 | .000 | .834 | 71 | .000 |
| I have sufficient behavioral science knowledge. | .232 | 71 | .000 | .851 | 71 | .000 |
| The level of my knowledge is sufficient | .224 | 71 | .000 | .864 | 71 | .000 |
| I felt prepared for clinical skill performance | .231 | 70 | .000 | .858 | 70 | .000 |
| There is a lot of learning from chairside teaching | .252 | 71 | .000 | .789 | 71 | .000 |
| The knowledge acquired in clinical is easier to remember | .258 | 71 | .000 | .807 | 71 | .000 |
| Clinical faculty are good teachers | .234 | 71 | .000 | .824 | 71 | .000 |
| I am able to judge my own progress | .255 | 71 | .000 | .835 | 71 | .000 |
| I study primarily to pass tests and examinations | .235 | 71 | .000 | .842 | 71 | .000 |
| What I study depends on the problems I face during my clinical exposure | .287 | 71 | .000 | .841 | 71 | .000 |
| Problem based learning ( PBL ) provided good preparation for clinical practice | .228 | 71 | .000 | .879 | 71 | .000 |
| I study more after starting my clinical training | .284 | 71 | .000 | .843 | 71 | .000 |
| My learning is influenced by the questions from clinical faculty | .289 | 71 | .000 | .843 | 71 | .000 |
| I feel the need to study because I have forgotten my theoretical knowledge | .206 | 71 | .000 | .854 | 71 | .000 |
| Pre-clinical courses were good preparation for learning skills which are applied in the clinics | .228 | 70 | .000 | .851 | 70 | .000 |
| a. Lilliefors Significance Correction | | | | | | |
